# Hybrid graphene metasurfaces for high-speed mid-infrared light modulation and single-pixel imaging

**DOI:** 10.1038/s41377-018-0055-4

**Published:** 2018-08-22

**Authors:** Beibei Zeng, Zhiqin Huang, Akhilesh Singh, Yu Yao, Abul K. Azad, Aditya D. Mohite, Antoinette J. Taylor, David R. Smith, Hou-Tong Chen

**Affiliations:** 1 Center for Integrated Nanotechnologies, Los Alamos National Laboratory, Los Alamos, New Mexico 87545 USA; 2 Center for Metamaterials and Integrated Plasmonics, Department of Electrical and Computer Engineering, Duke University, Durham, North Carolina 27708 USA; 3 MPA-11, Los Alamos National Laboratory, Los Alamos, New Mexico 87545 USA; 4 School of Electrical, Computer and Energy Engineering, Arizona State University, Tempe, Arizona 85287 USA; 5 Chemistry, Life, and Earth Sciences Directorate, Los Alamos National Laboratory, Los Alamos, New Mexico 87545 USA; 6 Department of Chemical and Biomolecular Engineering, Rice University, Houston, Texas 77005 USA

## Abstract

During the past decades, major advances have been made in both the generation and detection of infrared light; however, its efficient wavefront manipulation and information processing still encounter great challenges. Efficient and fast optoelectronic modulators and spatial light modulators are required for mid-infrared imaging, sensing, security screening, communication and navigation, to name a few. However, their development remains elusive, and prevailing methods reported so far have suffered from drawbacks that significantly limit their practical applications. In this study, by leveraging graphene and metasurfaces, we demonstrate a high-performance free-space mid-infrared modulator operating at gigahertz speeds, low gate voltage and room temperature. We further pixelate the hybrid graphene metasurface to form a prototype spatial light modulator for high frame rate single-pixel imaging, suggesting orders of magnitude improvement over conventional liquid crystal or micromirror-based spatial light modulators. This work opens up the possibility of exploring wavefront engineering for infrared technologies for which fast temporal and spatial modulations are indispensable.

## Metamaterials: mid-infrared modulator

Ultrafast modulators and spatial light modulators (SLMs) for mid-infrared light have been realized by the use of an electrically-tunable graphene on a silicon-integrated metasurface. The modulators designed and built by Beibei Zeng and coworkers from Los Alamos National Laboratory, Duke University and Arizona State University in the US offer a depth of modulation of 90% and a modulation speed exceeding 1 GHz. The modulator consists of a sandwich structure consisting of an array of gold nanoantennas, a layer of graphene, atop a thin dielectric film of Al_2_O_3_, an amorphous-Si layer and a rear reflector made of gold. The structure also features gate and drain electrodes to electrically tune the device. When constructed into a 6x6 pixel array the modulators allow the realization of a SLM for proof-of-principle high frame rate single-pixel imaging in the mid-IR.

## Introduction

Emerging infrared technologies lie at the core of advanced photonic research because of their great potential for numerous applications. Despite the tremendous efforts in the development of sources^1^ and detectors^2^, prevailing methods previously reported for free-space infrared light modulation, which is essential for many applications^3–5^, have suffered from drawbacks particularly in the modulation depth and speed. For example, solid-state infrared light modulators based on semiconductor quantum wells either have a small modulation depth or require sophisticated material growth and cryogenic temperatures^6,7^. In addition, state-of-the-art liquid crystal^8^ and micromirror^9^-based spatial light modulators (SLMs) suffer from limitations including slow modulation speeds of a few kilohertz and complex and expensive instrumentation. In this context, active metamaterials/metasurfaces composed of planar subwavelength resonators and reconfigurable functional materials/devices have provided an alternative approach^10^. In the microwave region, the integration of electronic devices (e.g., varactor diodes) has resulted in reprogrammable coding metasurfaces^11–13^. In the far-infrared region, the integration of metasurfaces and compound semiconductors (e.g., GaAs) has enabled the modulation of terahertz (THz) waves with high modulation depth and speed at room temperature^14–17^. However, such configurations are difficult to scale up to the mid- and near-infrared regions, where electronic devices fail to operate and the optical conductivity of conventional semiconductors can only be slightly adjusted^18,19^, resulting in limited dynamic modulations.

In contrast, graphene has demonstrated exceptional electrical and optical properties and a widely tunable electro-optical response through the entire infrared region^20–23^. In addition, the compatibility of graphene with CMOS processing makes it an extremely promising material for cost-effective optoelectronics in high-frequency applications^24,25^. Most recently, hybrid graphene metasurfaces have shown unprecedented capabilities in developing electrically reconfigurable infrared optoelectronic devices^26–30,46,47^, although in these designs the modulation relies on a high voltage bias and the modulation speed is limited, and many studies have focused on a waveguide configuration^22,25,31^. In this paper, we present the experimental demonstration of hybrid graphene metasurface free-space mid-infrared modulators that enable a large intensity modulation depth of up to 90% and a high modulation speed exceeding 1 GHz over a broad bandwidth by tuning the Fermi level of graphene at a low gate voltage bias of ∼7 V. The low-voltage operation achieved by the design architecture of our device is essential for obtaining a high-quality modulation signal and an ultra-high modulation speed under ambient conditions. Furthermore, a prototype mid-infrared SLM is demonstrated to function as an electrically encoded aperture or mask, realizing single-pixel imaging with a high frame rate.

## Results

**Fig. 1:**
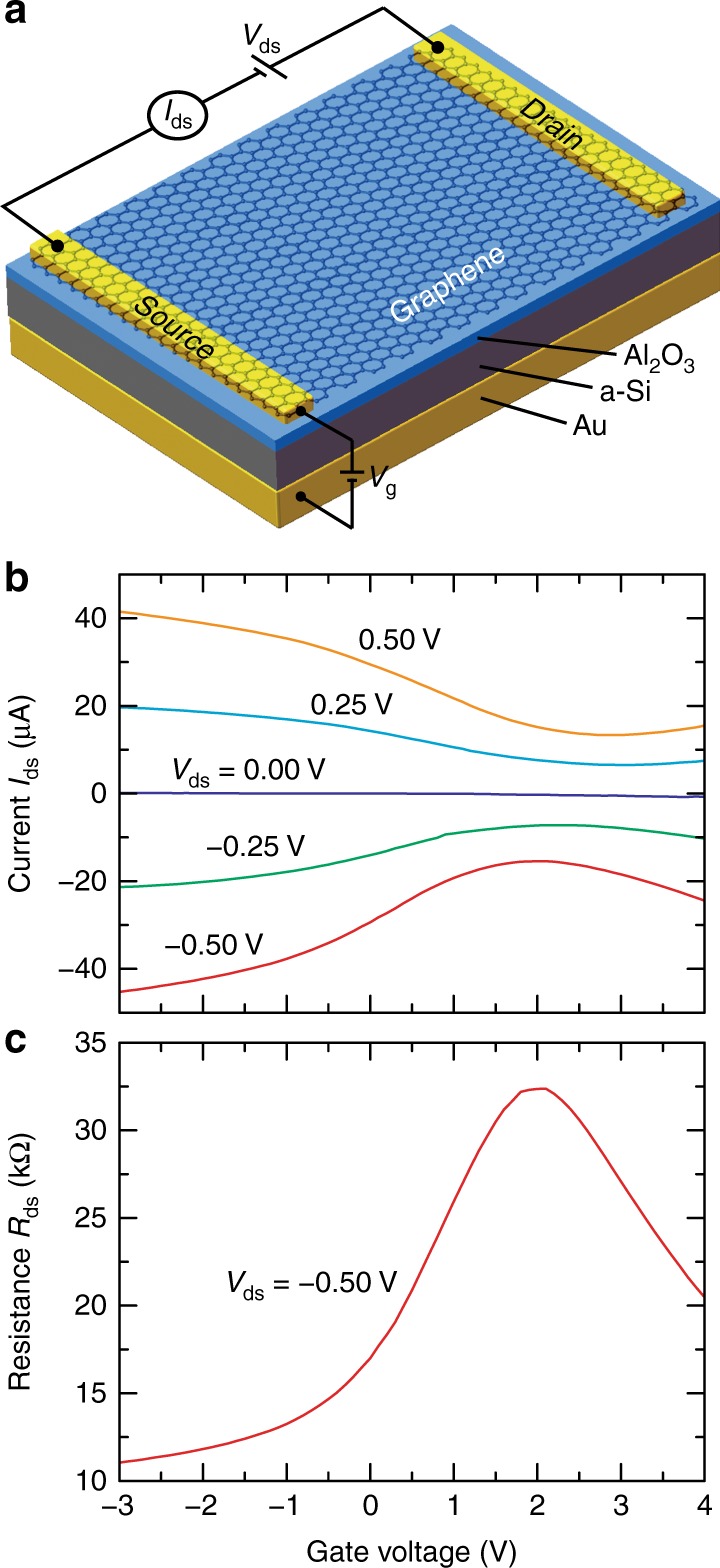
Tuning graphene conductivity with a low gate voltage bias. **a** Schematic of the FET structure in which a-Si serves as part of the back-gate electrode and the ultrathin Al_2_O_3_ layer serves as the gate dielectric. **b** Measured drain-source current *I*_ds_ across the graphene as a function of gate voltage *V*_g_ at different drain-source biases *V*_ds_. **c** Graphene resistance *R*_ds_ as a function of gate voltage at *V*_ds_ = −0.50V, revealing the graphene charge neutrality point at approximately 2 V

A major issue in frequency-tunable hybrid graphene metamaterial absorbers is the requirement of a high voltage bias^27–30,46,47^, which also limits the modulation speed and the scope of applications. Operating at mid-infrared wavelengths, the high gate voltage is due to the thick dielectric spacer (typically a few hundred nanometers) between the metal ground plane and metallic resonator array^32^. When graphene is integrated into the resonator array, the metamaterial absorber is essentially a field-effect transistor (FET) structure in which the application of a gate voltage *V*_g_ tunes the graphene conductivity by varying the Fermi level *μ*_C_ according to^21,33^ *μ*_C_*=* *ħv*(*πCV*_g_/*e*)^1/2^. To lower the gate voltage, it is necessary to increase the capacitance *C* by decreasing the gate dielectric thickness (i.e., the dielectric spacer in metamaterial absorbers). Although we cannot reduce the dielectric spacer thickness of a metamaterial absorber, an alternative solution is to replace a large portion of the dielectric spacer with a material that is conducting for the applied voltage bias but behaves as a dielectric in the mid-infrared range. This strategy is demonstrated by using the FET structure schematically shown in Fig. 1a, where the slightly conducting a-Si layer serves as part of the gate electrode, and the gate dielectric is provided by the ultrathin Al_2_O_3_ layer to increase the capacitance. In this way, we are able to significantly reduce the gate voltage to a few volts to reach the graphene charge neutrality point and effectively tune the graphene conductivity, as shown in Fig. 1b, c where we plot the drain-source currents *I*_ds_ and resistance *R*_ds_ as functions of gate voltage *V*_g_ for a few different drain-source biases *V*_ds_.

**Fig. 2:**
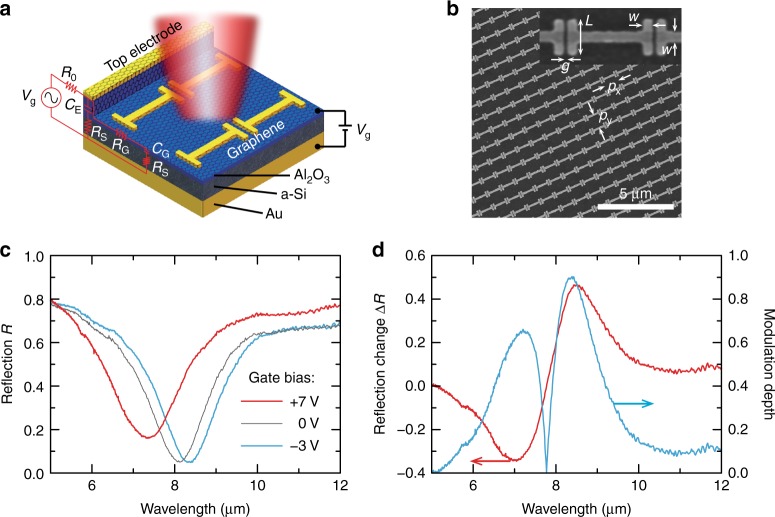
Hybrid graphene metasurface allows for electrically tunable resonant absorption. **a** Schematic of the hybrid graphene metasurface. Inset: high-frequency equivalent circuit model. **b** SEM image showing the fabricated gold nanoantenna array. Inset: zoom-in view. *P*_x_ = *P*_y_ = 1 μm, *L* = 400 nm, *w* = 100 nm, and *g* = 40 nm. **c** Measured reflection spectra when applying different gate voltages *V*_g_. **d** Change of reflection Δ*R*(*λ*) = *R*_+7 V_(*λ*) − R_−3 V_(*λ*) (red curve) and modulation depth *M*(*λ*) = |Δ*R*(*λ*)|/max[*R*_+7 V_(*λ*);*R*_−3 V_(*λ*)] (cyan curve) for two gate voltages of *V*_g_ = +7 V and −3 V as functions of wavelength *λ*

Following this strategy, our hybrid graphene metasurface structure is schematically shown in Fig. 2a, where the dielectric spacer is mainly formed by the a-Si layer. The metamaterial absorber can be considered as a subwavelength cavity formed between the nanoantenna array and the ground plane, where the dispersion of the nanoantenna resonant response results in a phase condition such that the multireflection experiences destructive interference, leading to the cancellation of the overall reflection and induction of high absorption over a narrow wavelength range^28,34^. That is, the incident light is trapped within the metamaterial cavity and eventually absorbed by the metallic structure, the lossy dielectric spacer, or both. Because the optical loss of a-Si is negligible in the mid-infrared region, the absorption mainly occurs via ohmic dissipation at the metallic resonator array integrated with graphene. These two facts give rise to the possibility of tuning the resonant dispersion of the nanoantenna array with the goal of satisfying the phase condition and thereby the absorption at a different wavelength by tuning the conductivity of the integrated graphene through the application of a gate voltage. In Fig. 2c, we plot the measured reflection spectra of the hybrid graphene metasurface (SEM image shown in Fig. 2b) when applying different gate voltages. They all show a deep reflection dip that suggests high absorption, as the transmission is zero. Upon applying the gate voltage bias, the resonance shifts from *λ* = 7.3 μm (reflection minimum ∼16%) to 8.3 μm (reflection minimum ∼5%) when the low gate voltage changes from +7 V to −3 V. The corresponding change of reflection ∆*R*(*λ*) = *R*_+7 V_(*λ*) − *R*_−3 V_(*λ*) and modulation depth *M* = |∆*R*(*λ*)|/max[*R*_+7 V_(*λ*), *R*_−3 V_(λ)] are plotted in Fig. 2d as functions of wavelength, revealing the maximum reflection change of 46.7% (from 53.1% at +7 V to 6.4% at −3 V) at *λ* = 8.5 μm and a modulation depth as high as ∼90% in this wavelength range.

**Fig. 3:**
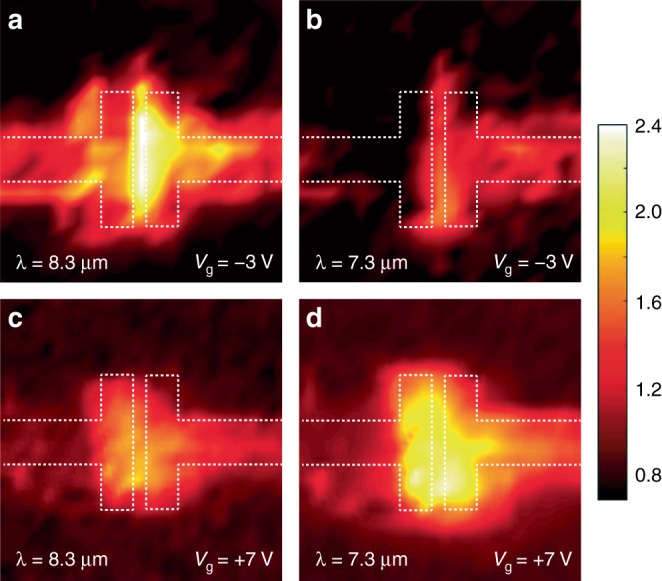
Near-field imaging of gate-tunable plasmonic modes. The near-field images are taken within a unit cell area using broadband illumination, under gate voltages of *V*_g_ = −3 V (**a**, **b**) and +7 V (**c**, **d**), and the images are extracted at *λ* = 8.3 μm (**a**, **c**) and 7.3 μm (**b**, **d**). White dashed lines mark the boundaries of the gold nanoantennas

The shifting of the plasmonic resonance dispersion can be visualized through near-field imaging of localized plasmonic modes in the hybrid graphene metasurface with different gate voltages^35,36^. The gate-tunable plasmonic modes are clearly shown in Fig. 3 within a unit cell of the hybrid graphene metasurface. When the applied gate voltage is −3 V, the electromagnetic field is highly confined and enhanced in the gap area between two nanoantennas, as shown in Fig. 3a, at *λ* = 8.3 μm, corresponding to the reflection minimum in Fig. 2c. The field confinement and enhancement are much less obvious at the off-resonance wavelength *λ* = 7.3 μm, as shown in Fig. 3b. In contrast, when the gate voltage is increased to +7 V, the enhanced field can be clearly seen at *λ* = 7.3 μm, as shown in Fig. 3d, rather than at *λ* = 8.3 μm, as shown in Fig. 3c.

**Fig. 4:**
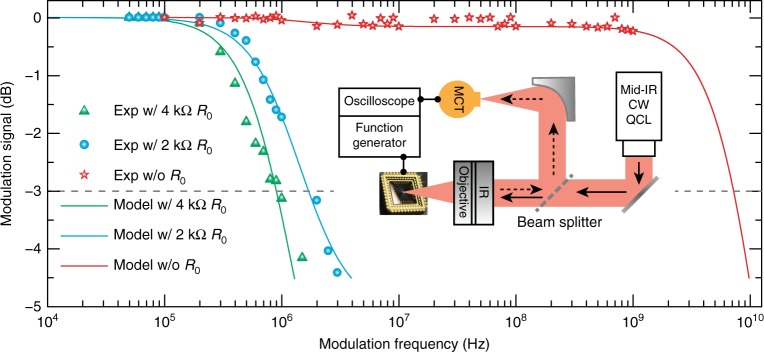
Modulation speed of the hybrid graphene metasurface. Symbols represent the measured modulation signal as a function of gate voltage frequency for the device with intrinsic modulation (red stars) and device loaded with an external resistor of *R*_0_ = 2 kΩ (cyan circles) or 4 kΩ (green triangles). The cyan and green curves represent the modulation fitting based on an equivalent circuit model when the metasurface modulator is loaded with *R*_0_ = 2 kΩ and 4 kΩ external resistors, respectively. The fitting parameters are then used to retrieve the intrinsic modulation characteristics of the metasurface modulator, represented by the red curve. Inset: schematic of the modulation speed measurement system

In addition to the high modulation depth, the hybrid graphene metasurface modulator enables an ultra-high modulation speed. Our characterization system, schematically shown in the inset to Fig. 4, allows one to measure only the modulation up to 1 GHz, where the modulation signal does not yet show any significant decay, as revealed by the red stars shown in Fig. 4. To quantify the modulation cut-off frequency, we purposely reduce the device operation speed by loading an external resistor^15^ with a resistance of either *R*_0_ = 2 kΩ or 4 kΩ. The cyan circles and green triangles in Fig. 4 represent the measured optical modulation signals as a function of the gate frequency with *R*_0_ = 2 kΩ and 4 kΩ external resistors, respectively, from which we can clearly observe the different modulation cut-off frequencies. Using the high-frequency circuit model^27^, the experimental data are fitted with a set of circuit parameters. The results are shown as the solid curves. Based on the same set of device circuit parameters, the intrinsic modulation is shown as the red curve in Fig. 4, retrieved by removing the external resistor. A 3 dB cut-off frequency of 7.2 GHz is inferred, which represents the fastest free-space mid-infrared optical modulation to date using an electrical approach. In addition to the low-voltage operation, this high modulation speed is attributed to the reduction of the electrical contact area and the thickness increase of the insulating layer underneath the top electrode, both of which significantly reduce the parasitic capacitance of the device.

**Fig. 5:**
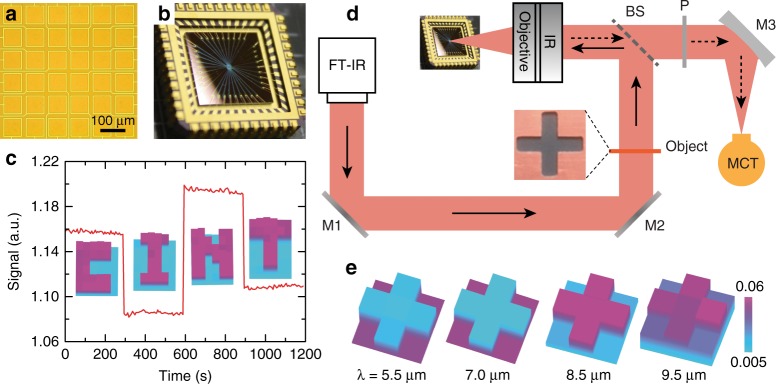
Hybrid graphene metasurface spatial light modulator. **a** An optical micrograph showing the active area consisting of an array of 6 × 6 functional pixels. **b** A photograph of the device after being wire-bonded to a chip carrier. **c** Spatial reflection patterns of “CINT” at *λ* = 8.3 μm by selectively applying gate voltages *V*_g_ = −3 V (‘OFF’) and +7 V (‘ON’) to individual pixels in accordance with the patterns shown by the images in the insets (purple color indicates ‘ON’). Changes among SLM patterns result in different measured signal intensities from the single-pixel detector. The images in the insets are taken by raster-scanning pixels, i.e., the corresponding pixel is either ‘ON’ or ‘OFF’ while all other pixels remain ‘OFF’. **d** Schematic of the single-pixel imaging setup employing the hybrid graphene metasurface spatial light modulator. M1 and M2 flat mirrors, BS beam splitter, P polarizer, M3 parabolic mirror, and MCT mercury-cadmium-telluride single-pixel detector. **e** Reconstructed images of a cross-shaped object using a raster-scan measurement matrix at different wavelengths of *λ* = 5.5 μm, 7 μm, 8.5 μm, and 9.5 μm

The high modulation depth and fast modulation speed make the hybrid graphene metasurface an ideal platform to create SLMs for wavefront coding in mid-infrared applications, such as high frame rate single-pixel imaging, where the traditional liquid crystal and micromirror-based SLMs suffer from limitations in modulation speed. In the fabricated metasurface SLM prototype device consisting of 6 × 6 functional pixels shown in Fig. 5a, b, the electrically isolated functional pixels allow us to independently switch ‘ON’ or ‘OFF’ the individual pixels by applying a gate voltage of +7 V or −3 V, respectively. We first characterize its operation by raster scanning the functional pixels—measuring the reflection intensity as we turn only one pixel ‘ON’ or ‘OFF’ following the mask patterns of the four letters in “CINT”, while all other pixels remain ‘OFF’. The corresponding results are shown in the insets to Fig. 5c, clearly visualizing the mask patterns measured at the wavelength of *λ* = 8.3 μm. By simultaneously turning on the functional pixels in the corresponding mask patterns, the red curve in Fig. 5c plots the real-time reflected light signal that displays a variation upon changing to different mask patterns. Most of the temporal noise is caused by the Globar infrared light source. The single-pixel imaging capability of the hybrid graphene metasurface SLM was demonstrated using the setup schematically shown in Fig. 5d. In Fig. 5e, we show exemplary reconstructed images of a cross-shaped object at different wavelengths, demonstrating the broadband single-pixel imaging capability. A high image contrast is observed in the middle two panels for *λ* = 7 μm and 8.5 μm due to their excellent modulation depth, in contrast to the lower imaging contrast at *λ* = 5.5 μm and 9.5 μm due to their reduced modulation depth (see Fig. 2d). The frame rate (speed of image acquisition) of the single-pixel imaging system can be estimated to be 23 kHz, which is more than two orders of magnitude faster than the value of 50 Hz for state-of-the-art infrared cameras, assuming that they have the same imaging resolution (640 × 480). Our result can be further improved by utilizing compressive imaging techniques^37,38^. Thus, this imaging system is suitable for the detection of transient thermal phenomena and holds great potential for security screening, navigation, and medical detection^3,4^.

## Discussion

We have demonstrated a hybrid graphene metasurface modulator for free-space mid-infrared light based on a tunable metamaterial absorber design. With a gate bias of a few volts to tune the graphene conductivity, the room temperature device achieves high modulation depths of up to 90% and a modulation speed exceeding 1 GHz, which is the fastest free-space mid-infrared modulator to the best of our knowledge. The intrinsic modulation speed was inferred to be approximately 7 GHz, which could be further improved by reducing the device active area or the pixel size in the SLMs. The low-voltage operation is attributed to the reduction of gate capacitance by effectively decreasing the gate dielectric thickness through the replacement of a large portion of the dielectric material with a semiconducting material (a-Si) in the spacer of the metamaterial absorber. For the mid-infrared light, the a-Si layer can be considered as a dielectric material with negligible loss, but the semiconducting properties make it suitable to serve as part of the electrode for applying a gate voltage bias to tune the graphene conductivity, resulting in shifted/damped resonant dispersion of the nanoantenna array and thus frequency tuning of the metasurface absorption. The high-speed modulation is in part due to the low voltage operation and the significant reduction of parasitic capacitance from the electrodes.

The hybrid graphene metasurface modulator was then formed into a pixel array to serve as a prototype mid-infrared SLM. We showed that single-pixel imaging can be achieved by creating a series of spatial mask patterns, revealing orders of magnitude faster imaging speeds than those obtained using traditional liquid crystal and micromirror spatial light modulators. The speed of single-pixel imaging can be further enhanced by adopting a compressive single-pixel imaging algorithm^37,38^. This work opens up the possibility of exploring wavefront engineering for infrared technologies for which fast temporal and spatial modulations are indispensable, and the demonstrated high frame-rate imaging approach holds great potential for many applications, such as the detection of transient thermal phenomena, real-time thermal imaging, security screening, navigation, and medical detection.

We note that the optics in Fig. 5d for casting the image of an object onto the SLM shares the same working principle as a camera or a telescope equipped with a sensor array to record or an eyepiece to magnify the image. Therefore, the quality and field of view of the image are essentially determined by this part of the optics. The rest of the optical path is basically a microscope (with the same objective) to capture the SLM-coded image using the single-pixel detector rather than a microscope eyepiece. To recover the image of a more complex object using compressive single-pixel imaging algorithms, in the future, we must increase the area and pixel number of the SLM and reduce the pixel size to the scale of the operational wavelength. In this case, the current design of top electrodes becomes impractical, and the SLM must be redesigned to allow for a large array of back gates^39^. We would like to emphasize that the prototype mid-infrared SLM can be scaled to operate at other frequencies, including terahertz, near-infrared, and even visible, since graphene has been proven to maintain the high optical conductivity and have opto-electronic responses at these frequencies due to the intra-band (terahertz and mid-infrared) and inter-band (near-infrared and visible) transitions^22,23,40–42^. The basic principle is the same, but when approaching shorter wavelengths, the concern is the increasing absorption in the amorphous silicon spacer layer, which can potentially reduce the modulation depth. Additionally, when scaling to operate at shorter wavelengths, the further reduced critical dimension of the metasurface structure and back gate array can potentially create fabrication challenges.

## Materials and methods

### Structure design and fabrication

Figure 1a schematically illustrates the field-effect transistor (FET) structure for tuning graphene conductivity, which consists of a 150 nm-thick Au ground plane by e-beam evaporation, a 400 nm-thick e-beam-evaporated amorphous silicon (a-Si) layer with a 1 nm-thick Ti adhesion layer also by e-beam evaporation, a 6 nm-thick ultrathin Al_2_O_3_ gate dielectric grown by atomic layer deposition (ALD), two photolithography-defined 150 nm-thick gold strips serving as source and drain electrodes, and a transferred monolayer graphene grown by low-pressure chemical vapor deposition (LPCVD). Figure 2a depicts the device schematic of our hybrid graphene metasurface modulator operating in reflection mode, which is based on a modified metamaterial perfect absorber design^28,43^. Compared to the FET structure shown in Fig. 1a, the source and drain electrodes were replaced by a 6 × 6 array of top rectangular-loop-shaped electrodes each surrounding a square active area of 75 μm × 75 μm (see Fig. 5a), which were defined before the graphene transfer using standard photolithographic methods followed by e-beam deposition of a 750 nm-thick Al_2_O_3_ insulating layer and 3 nm/30 nm Ti/Au layers and a lift-off process. Graphene was then transferred and patterned to align with the electrode array, with graphene between electrodes removed by photolithography and O_2_ plasma etching, defining electrically isolated functional pixels as depicted by the optical micrograph in Fig. 5a for a completed device. An array of 30 nm-thick ‘I’-shaped Au nanoantennas with a 1.5 nm-thick Ti adhesion layer was finally fabricated within each pixel using standard e-beam lithography methods, metal deposition, and a lift-off process, with a scanning electron microscopy (SEM) image shown in Fig. 2b. Upon the completion of sample fabrication, the sample was diced into a 9 mm × 9 mm chip and wire bonded to external pins of a chip carrier through a predefined electrode array, as shown in Fig. 5b.

### Graphene growth and transfer

Graphene was grown on a piece of copper foil (0.025 mm thick, 99.8%, Alfa Aesar) in a home-built LPCVD system^44,45^. The copper foil was first loaded in a quartz tube and annealed at 1000 °C for 1 h while maintaining the H_2_/Ar pressure at 1 Torr under a 200 sccm flow. Graphene was then grown for 30 min by introducing 50 sccm CH_4_ at a total pressure of 1.7 Torr. The furnace was then rapidly cooled to room temperature. The growth conditions outlined above produced a large-area, continuous graphene film with very few multi-layer regions or cracks. To transfer the graphene layer to the substrate, PMMA (950 A4) was spin-coated onto one side of the graphene/copper/graphene foil and stored in vacuum overnight. The graphene on the backside was then removed with O_2_ plasma etching. The sample was then placed in an FeCl_3_-based etchant for 2 h, allowing the copper to completely dissolve. The remaining graphene/PMMA film was cleaned in HCl solution, rinsed with DI water, transferred onto the substrate, and put into acetone to remove the PMMA layer.

### Device characterization

Each pixel of the 6 × 6 pixel array was independently connected to one of the 36 channels (output voltage, −4V ∼+8 V) of a DAC evaluation board (EVAL-AD5370, Analog Devices Inc.), which was then controlled by a microprocessor (Raspberry Pi 3, Raspberry Pi Foundation) using home-built Python codes. The reflection spectra of the individual pixels under different voltage biases were measured using a Fourier transform infrared spectroscopy microscope (FTIR, Bruker). The plasmonic resonance modes were visualized using a scattering-type near-field scanning optical microscope (s-NSOM, neaSpec)^35,36^ equipped with a broadband infrared beam by difference frequency generation (DFG) to cover a wide mid-infrared range from 650 to 2400 cm^−1^. The raw data contain spectral information due to the use of broadband illumination, and we used Matlab programming to extract near-field images at specific wavelengths. The modulation speed was measured for individual pixels using the setup schematically shown in the inset to Fig. 4. We used a tunable continuous-wave quantum cascade laser (CW-QCL, MIRcat-1100, Daylight Solutions) light source and a thermoelectrically cooled mercury cadmium telluride (TE-MCT, PVI-2TE-10.6, VIGO System S.A.) detector. The mid-infrared light (*λ* = 6.75 μm) was transmitted through a beam splitter (55/45) and focused onto the hybrid graphene metasurface modulator with a mid-infrared objective. The reflected light was collected by the same objective, reflected by the beam splitter, and focused into the TE-MCT detector using an off-axis parabolic mirror. A functional pixel of the device was applied with  a variable frequency sine voltage with offset 0 V and amplitude 1 V from a function generator integrated with an oscilloscope (Tektronix, MDO 3104). The response of the TE-MCT detector was measured with the oscilloscope, determining the optical modulation signal of the device. Note that we were only able to measure the modulation frequency up to 1 GHz due to the limited frequency range of the function generator and TE-MCT detector and the low signal-to-noise ratio of our measurement setup. Reduction of the modulation cut-off frequency (at 3 dB) within the measurement range was achieved by inserting different series resistors (e.g., *R*_0_ = 2 kΩ and 4 kΩ) into the driving circuit^15^. The intrinsic modulation speed was then inferred using a high-frequency circuit model of the device. The spatial light modulation was characterized using the FTIR microscope by raster scanning the functional pixels—measuring the reflection intensity using a single-pixel detector as we turned only one pixel ‘ON’ or ‘OFF’ while all other pixels remained ‘OFF’. The imaging capability of the hybrid graphene metasurface SLM was demonstrated using the setup schematically shown in Fig. 5d. Mid-infrared light from the Globar broadband source illuminates a cross-shaped object, and the image (*X*) is projected onto the SLM through an infrared objective lens. The reflected light thus represents the image encoded by the SLM mask (forming the measurement matrix *Φ*) and is collected by the same objective and focused onto the MCT single-pixel detector (*Y*). For a proof-of-concept demonstration, a raster-scan measurement matrix *Φ* of the SLM mask was employed by sequentially turning on/off only one pixel for each measurement, resulting in a 36 × 36 identity matrix *Φ*. The original image *X* was then reconstructed by *X* *=* *Φ*^−1^*Y*, where *Φ*^−1^ is the inverse measurement matrix.

### High-frequency circuit model

A simplified high-frequency circuit model of the device was developed, as shown in the inset to Fig. 2a, where *C*_E_ (*C*_G_) represents the capacitance between the top Au electrode (graphene) and the a-Si layer across the 750 nm-thick (6 nm-thick) Al_2_O_3_ insulating layer, *R*_G_ and *R*_S_ are the resistances of the graphene and a-Si, respectively, and *R*_0_ is the externally loaded resistance. The high-frequency modulation voltage from a function generator was applied to the device as the gate voltage *V*_g_(*ω*), and the modulation voltage *V*_m_(*ω*) at the functional pixel and the frequency-dependent modulation depth *η*(*ω*) of the device were derived as

1 $$V_{\rm m}\left( \omega \right) = \eta \left( \omega \right)V_{\rm g}\left( \omega \right)$$

2 $$\eta \left( \omega \right) = {\textstyle{1 \over {1 + i\omega C_{\rm G}\left( {R_{\rm S} + R_{\rm G}} \right)}}} \times {\textstyle{{\left( {R_{\rm S} + R_{\rm G} + \frac{1}{{i\omega C_{\rm G}}}} \right)\left( {R_{\rm S} + \frac{1}{{i\omega C_{\rm E}}}} \right)} \over {\left( {R_{\rm S} + R_{\rm G} + \frac{1}{{i\omega C_{\rm G}}}} \right)\left( {R_{\rm S} + \frac{1}{{i\omega C_{\rm E}}}} \right) + R_0\left[ {\left( {R_{\rm S} + R_{\rm G} + \frac{1}{{i\omega C_{\rm G}}}} \right) + \left( {R_{\rm S} + \frac{1}{{i\omega C_{\rm E}}}} \right)} \right]}}}$$
